# Transcription Activator Swi6 Interacts with Mbp1 in *Mlu*I Cell Cycle Box-Binding Complex and Regulates Hyphal Differentiation and Virulence in *Beauveria bassiana*

**DOI:** 10.3390/jof7060411

**Published:** 2021-05-25

**Authors:** Jin-Li Ding, Jia Hou, Xiu-Hui Li, Ming-Guang Feng, Sheng-Hua Ying

**Affiliations:** Institute of Microbiology, College of Life Sciences, Zhejiang University, Hangzhou 310058, China; 11907002@zju.edu.cn (J.-L.D.); 12107004@zju.edu.cn (J.H.); 219070242@zju.edu.cn (X.-H.L.); mgfeng@zju.edu.cn (M.-G.F.)

**Keywords:** MBF complex, development, conidiation, dimorphism, pathogenicity, insect myco-pathogen

## Abstract

Mbp1 protein acts as a DNA-binding protein in *Mlu*I cell cycle box-binding complex (MBF) and plays an essential role in filamentous myco-pathogen *Beauveria bassiana*.In the current study, BbSwi6 (a homologue of yeast Swi6) was functionally characterized in *B.bassiana*. Both BbSwi6 and BbMbp1 localize in the nucleus and display a direct interaction relationship which is indicated by a yeast two-hybrid assay. *BbSwi6* significantly contributes to hyphal growth, asexual sporulation and virulence. On the aerial surface, Δ*BbSwi6* grew slower on various nutrients and displayed abnormal conidia-producing structures, which hardly produced conidia. In liquid media, *BbSwi6* loss led to 90% reduction in blastospore yield. Finally, the virulence of the Δ*BbSwi6* mutant was modestly weakened with a reduction of 20% in median lethal time. Comparative transcriptomics revealed that BbSwi6 mediated different transcriptomes during fungal development into conidia and blastospores. Notably, under the indicated condition, the BbSwi6-mediated transcriptome significantly differed to that mediated by BbMbp1. Our results demonstrate that, in addition to their roles as the interactive components in MBF, BbSwi6 and BbMbp1 mediate divergent genetic pathways during morphological transitions in *B. bassiana*.

## 1. Introduction

Filamentous fungi have evolved a conidiation process to produce numerous conidia for dispersal and survival in their environment [1]. *Beauveria bassiana* is a filamentous entomopathogenic fungus that naturally parasitizes insects, and therefore has a great potential for pest management [2]. Conidia attach to the host cuticle and germinate into invasive mycelia via mobilizing endogenous nutrients, which initiates the infection process [3,4]. Once entering into the insect hemocoel, *B. bassiana* develops into yeast-like cells (in vivo hyphal bodies) via dimorphic transition. Hyphal bodies assimilate the nutrients from hemolymph and propagate rapidly. At the late stage of infection, hyphal bodies transform into robust mycelia which cause the host to be mummified [5]. Finally, fungal hyphae grow outside of the cadaver and generate numerous conidia for the sequential infection cycle [6,7]. Therefore, conidiation process is critical for the consecutive infection cycle of *B. bassiana*. The cell-division cycle is essential for fungal proliferation [8]. Previous studies have revealed that the cell-division cycle mechanism is involved in development and morphological transition during *B. bassiana* lifecycle [9,10].

In *Saccharomyces cerevisiae*, this cycle included four phases, i.e., genome duplication (S-phase), nuclear division (M-phase) and two gap phases (G1 and G2). Two transcription-factor complexes (Swi4/Swi6 (SBF) and *Mlu*I (MBF) cell cycle box binding complex) possess comprehensive transcriptional regulation at G1/S transition (a checkpoint of cell-division cycle) [11,12]. In *S. cerevisiae* SBF and MBF, Swi6 acts as a transcriptional activator and recruits Swi4 and Mbp1, respectively [13,14]. However, the MBF components differ between yeast species. In *Candida albicans*, Swi4 and Swi6 are necessary for the phase transition of G1/S, but SBF lacks the binding sites in the genome. Hence, these two factors may be involved in MBF activity [15]. In *Schizosaccharomyces pombe*, MBF activates transcription of genes associated with G1/S transition and consists of Cdc10 (the ortholog of *S. cerevisiae* Swi6) and two DNA binding factors (i.e., Res1 and Res2) [16,17]. Furthermore, the MBF components play different roles in fungal physiology. Inbudding yeast, although cells without Mbp1 or Swi4 are nonviable, the Swi6 loss does not influence cell viability [11,12]. Our recent study has revealed that Mbp1 significantly contributes to the conidiation process in *B. bassiana*, promoting the understanding of the Mbp1 roles in filamentous fungi [10]. In the filamentous fungi *Magnaporthe oryzae* and *Fusarium graminearum*, Swi6 functions in growth as well as in both sexual and asexual development [18,19]. In *Metarhizium rileyi* (also an insect pathogenic fungus), the role of Swi6 in fungal conidiation has been examined [20], but the functions of the Swi6 homolog remain largely unknown in other entomopathogenic fungi. Although Mbp1 and Swi6 have some overlaps in fungal development and virulence, the question regarding their differential regulatory functions is still open.

To improve understanding of the differential roles of the different MBF components in the filamentous fungi, *B. bassiana* was used a representative organism. In this study, *B. bassiana* Swi6 (BbSwi6) was identified and verified as interacting with BbMbp1. Functional analyses indicated that *BbSwi6* was essential for fungal development and virulence in *B. bassiana*. During the conidiation and blastospore production process, comparative transcriptomic analysis was used to reveal the BbSwi6-mediated transcriptome which partially overlaps with the BbMbp1-mediated one [10]. We further found that the overlapped downstream 928 genes played an important role in aerial conidiation and 520 genes played a significant role in blastospore production. Our results highlight that, in addition to acting as the MBF components, Swi6 and Mbp1 display significantly functional differentiation during fungal development.

## 2. Materials and Methods

### 2.1. Strains and Growth Conditions

Fungal strains were cultured as previously described [10]. The wild-type *B. bassiana* ARSEF2860 (WT) (USDA-ARS Collection of Entomopathogenic Fungal Cultures, Ithaca, NY, USA)was cultivated on Sabouraud dextrose agar (SDAY: 4% glucose, 1% peptone and 1% yeast extract plus 1.5% agar). *Escherichia coli* DH5*α* (Invitrogen, Carlsbad, CA, USA) was cultured in Luria-Bertani (LB) medium for propagation of plasmids. *Agrobacterium tumefaciens* AGL-1 was cultured in YEB broth (*w*/*v*: 0.5% sucrose, 1% peptone, 0.1% yeast extract and 0.05% MgSO_4_) and used in fungal transformation. Czapek-Dox agar (CZA) (3% glucose, 0.3% NaNO_3_, 0.1% K_2_HPO_4_, 0.05% KCl, 0.05% MgSO_4_ and 0.001% FeSO_4_ plus 1.5% agar) was used to screen the transformants and phenotypic assay.

### 2.2. Bioinformatic Identification and Functional Analyses of BbSwi6

The sequence of *S. cerevisiae* Swi6 (GenBank No.: AJV59711) was used as a query to identify the homolog in the *B. bassiana* genome [21]. The resulting *B. bassiana* homolog (Locus tag: BBA_05428) was designated as BbSwi6, and then its entire ORF as well as the up- and downstream flanking sequences were identified by mapping the cDNA sequence of *BbSwi6* onto the fungal genomic sequence.

Fluorescence-coupled double screening method was used in gene disruption and complementation [22,23]. All primers are given in Table S1. The up- and downstream fragments were amplified by primer P1/P2 and P3/P4, respectively, and individually cloned into the *Xma*I/*Bam*HI sites and *Xba*I/*Spe*I sites in plasmid p0380-GTB in which *bar* cassette confers ammonium glufosinate resistance. The restriction enzymes were purchased from New England BioLabs, Inc. (Beijing, China). The resultant plasmid was named p0380-BbSwi6-KO, which was transformed in the WT strain. The 8-d old conidia were used as the receptor cells in fungal transformation. The transformants were grown on CZA supplemented with phosphinothricin (200 μg/mL) (45520, Sigma, MO, USA). The putative gene disruption mutant was screened by PCR reaction with primer P5 and P6, and was further verified under a laser scanning confocal microscope [23]. For gene complementation, the entire *BbSwi6* together with the promoter and terminator regions was amplified with the primer P7 and P8. The DNA fragment was cloned into the vector p0380-sur-gateway (conferring resistance to chlorsulfuron (C11325000, Dr. Ehrenstorfer GmbH, Augsburg, Germany)), generating the plasmid p0380-sur-BbSwi6. The resultant transformants were screened by PCR with the primer pair P5/P6 and further confirmed by qRT-PCR with the primer pair P9/P10.

### 2.3. Sub-Cellular Localization of BbMbp1 and BbSwi6

The intracellular locations of these two proteins were determined by fusing their nuclear location signal sequences to the fluorescent protein gene. Primers are listed in Table S1. Firstly, the promoter region of translation elongation factor 1 alpha gene (*TEF1*) was amplified with primer pair P11/P12. The DNA fragment was cloned into the *Eco*RI/*Bam*HI sites of p0380-sur [24], generating p0380-TEF-sur. Then, green fluorescent protein gene (GFP) fragments were amplified with the primer pair P13/P14 and cloned into the *Bam*HI sites of p0380-BbTEF-sur, generating pBbTEF-MCS-Gfp-sur (pBMGS). Similarly, the sequence of *mCherry* was amplified with the primer pair P15/P16, and cloned into the *Bam*HI sites of p0380-BbTEF-sur, generating pBbTEF-MCS-mCherry-sur (pBMRS).

The nuclear location signal sequences were predicted by DTU Bioinformatics (http://www.cbs.dtu.dk/services/) (accessed on 6 September 2020). The first 645 bp of *BbMbp1* were amplified with primer pair P17/P18, and cloned into pBMGS, generating pBMGS-BbMbp1. Similarly, the first 891 bp of *BbSwi6* were amplified with primer pair P19/P20, and integrated into pBMRS, generating pBMRS-*BbSwi6*. The resulting constructs were individually transformed into the WT strain. The strains expressing these fused genes were grown in Sabouraud dextrose broth (SDB). Two days later, fluorescent signals in mycelia were examined with a fluorescence microscope. Nuclei were stained with 4′,6-diamidine-2′-phenylindole dihydrochloride (DAPI) (D9542, Sigma, MO, USA).

### 2.4. Quantification of Blastospore Production

Phenotypic assay was conducted among the WT, gene disruption and complementation strains as described previously [10]. The blastospore formation was evaluated in SDB medium under submerged condition at 25 °C. Mycelia of the indicated strain were cultured in SDB medium and the resultant spores were used as initial inocula. Blastospore suspension was inoculated into fresh SDB medium and the final concentration was adjusted to 10^6^ spores/mL. Three days later, spore concentration in broth was quantified and blastospore yield was shown as spore number per milliliter.

### 2.5. Assay for Conidial Production

Conidiation on aerial surfaces was examined as described previously [10]. Inocula (in vitro blastospore) were prepared by culturing mycelia in SDB at 25 °C for 2 d. Aliquots of 100 μL blastospore suspension (10^7^ spores/mL) were cultured on SDAY plates and incubated at 25 °C. Mycelial discs were sampled at 4, 6 and 8 d post incubation (DPI). The conidia on mycelia were quantified and calculated as conidial number per cm^2^. In addition, the 3- and 8-d old mycelia were dried and weighed. The dry weight was presented as milligrams per square centimeter.

### 2.6. Fungal Growth

To evaluate the nutrient effects on vegetative growth, the carbon and nitrogen sources in CZA were replaced as necessary. Carbon sources (at a final concentration of 3%) included glucose, sucrose, trehalose and glycerol. Nitrogen sources (at a final concentration of 0.5%) included NH_4_Cl, NH_4_NO_3_, peptone and gelatin. One microliter of blastospore suspension (10^6^ spores/mL) was inoculated on the modified CZA plates. After 7 days of incubation, the colony diameter was measured.

### 2.7. Insect Bioassay

Fungal virulence against *Galleria mellonella* larvae (bioassay insect) was evaluated with an intra-hemocoel injection method. The insects were fed with artificialdiet and reared as previously described [25], and last-instar larvae (~300 mg in weight) were used. Aliquots of 5-μL of blastospore suspension (10^5^ spores/mL) were injected into the larval hemocoel. The experiment included three replicates, and approximately 35 larvae were used in every replicate. Control group was injected with Tween-80 solution (0.02%). The mortality was recorded daily and subjected to Probit analysis for the median lethal time (LT_50_). Cadavers were kept in a moisture chamber, and mycose on cadaver was recorded at 4 d post incubation.

### 2.8. Yeast Two Hybrid (Y2H) Assays

Protein-protein interactions were assayed with the Matchmaker^®^ Gold Yeast Two-Hybrid System (TaKaRa, CA, USA). All primers were listed in Table S1. The BbMbp1 ORF was amplified from cDNA template with primers P21/P22 and cloned into the pGADT7 vector. The resulting vector was confirmed by sequencing and transformed in pairs into yeast strain Y187 that was screened on selective (SD/-Leu) medium. The BbSwi6 ORF was obtained by PCR amplification with primer pair P23/P24 and cloned into the pGBKT7 vector. The vector was transformed into yeast strain Y2Gold and screened on selective (SD/-Trp) medium. These two strains were mated in YPDA medium and screened on the selective dropout/-tryptophane-leucine-adenine-histidine medium (SD-Trp-Leu-His-Ade) containing 200 ng/mL aureobasidin A (AbA) (600466, TaKaRa, Dalian, China).

### 2.9. Analyzing the BbSwi6-Mediated Transcriptome during Sporulation

All methods for transcriptomic analysis werethe same as those used in revealing the BbMbp1-mediated transcriptome [10]. Due to the presence of the transcriptome in the WT strain, only Δ*BbSwi6* mutant strain was subjected to RNA-seq analysis. Blastospores were prepared as mentioned above and used as initial inocula. Aerial mycelia were cultured on SDAY plates for 3 d, and submerged mycelia were grown in SDB medium for 2 d. Total RNA was extracted from the mycelial sample (Trizol method). The mRNA molecules were purified byusing magnetic oligo(dT) beads and used as templatesto synthesize first-strand cDNA using random hexamerprimers. Second-strand cDNA synthesis was performed in reaction buffer containing deoxyribonucleotide triphosphates and DNA polymerase I. Double-strand DNA was purified and constructed into a library which was analyzed on the Illumina HiSeq X Ten platform at Vazyme Biotech Co., Ltd. (Nanjing, Jiangsu, China). The sequence data have been deposited in the NCBI Gene Expression Omnibus (accession No. GSE171375). Two independent libraries were constructed for each treatment.

All clean reads were mapped onto the Bb2860 genome using the HISAT program [26]. The resultant genes were normalized in terms of the expected number of fragments per kilobase of transcript sequence per millions base pairs sequenced (FKPM), using Cufflinks software [27]. The transcriptomic data for the WT strain were downloaded from Gene Expression Omnibus (accession No.: GSE134764) [10]. The Cuffdiff method was used to search the differentially expressed genes (DEGs) between the WT and Δ*BbSwi6* mutant strains. The DEGs were considered when an absolute value of log_2_Ratio (fold change) > 1 at the threshold of the *q*-value < 0.05 was obtained (5% false discovery rate) [28]. The resulting DEGs were subjected to enrichment analysis, using the online FungiFun2 portal (https://elbe.hki-jena.de/fungifun/) (accessed on 6 September 2020). The threshold of the corrected *p*-value was set at 0.05 [29].

To determine whether *BbMbp1* and *BbSwi6* have different transcriptional influences during conidiation, we compared the BbSwi6- and BbMbp1-mediated DEGs [10].

### 2.10. Data Analyses

All measurements for the WT, gene disruption and complementation mutant strains, including the colony diameterand sporulation capacitywere subjected to two-way ANOVA. The measurements for LT_50_ were subjected to one-way ANOVA. Significant differences were determined with Tukey’s honest significance test (Tukey’s HSD).

## 3. Results

### 3.1. Bioinformatic Analysis of BbSwi6 and Generation of Its Gene Disruption and Complementation Strains

Based on a BLAST search with a yeast homolog as the query, a single highly related *B. bassiana* gene, BBA_05428 (identity: 28.80%; E-value: 7 × 10^−65^), was identified and named *BbSwi6*. The open reading frame (ORF) sequence of this gene was 2433 bp long and had one intron. *BbSwi6* coded for a protein with 810 amino acids.

To further examine *BbSwi6* roles, the gene disruption strain was successfully constructed through the homologous recombination strategy, and the partial ORF of *BbSwi6* was replaced by the phosphinothricin resistance gene (*bar*) (Figure S1A). To construct the complementation strain, the whole *BbSwi6* ORF plus the promoter region was ectopically integrated into the *BbSwi6* mutant strain, using the sulfonylurea resistance gene (*sur*) as the selection marker. The resulting disruption and complementation mutant strains were first screened by PCR and further verified by fluorescence detection and qRT-PCR analysis (Figure S1B–D).

### 3.2. BbSwi6 Interacts with BbMbp1 in Nucleus

**Figure 1:**
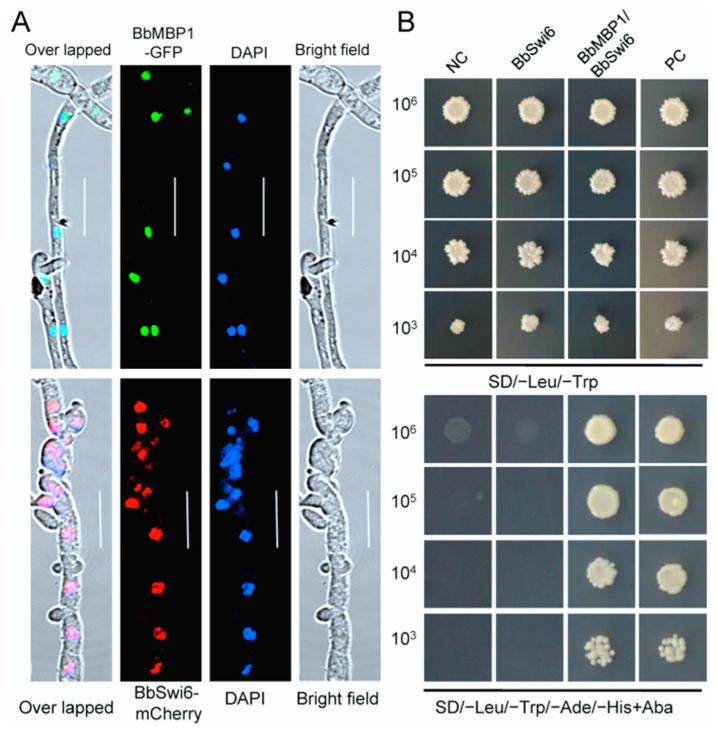
Sub-cellular analyses of the Swi6 and Mbp1 protein in *B. bassiana*. (**A**) The targeting signal sequences of Swi6 and Mbp1 were fused to *mCherry* and green fluorescent protein gene (*GFP*), respectively. The hybrid genes were transformed into the wild-type strain.Mycelia were stained with nucleus-specific dye DAPI. The overlapped fluorescent signals indicated that both Swi6 and Mbp1 localize in nucleus. (**B**) Yeast two-hybrid test indicated that Swi6 and Mbp1 have physical interaction. Only yeast cells with Swi6 and Mbp1 grew the selective media well. Negative and positive controls (NC and PC) were provided by kit. Scale bar: 10 μm.

Intracellular localization of BbSwi6 and BbMbp1 in *B. bassiana* was validated by fusing the coding sequence for nuclear targeting signal to the N-terminus of mCherry and green fluorescent protein gene (*GFP*), respectively. Nucleus was visualized by blue fluorescence using 4′,6-diamidino-2-phenylindole (DAPI, a nucleus-specific dye). Under a laser scanning confocal microscope, globular red and green signals were seen in the transformant expressing the fusion gene and were co-localized with the blue fluorescence well (Figure 1A). These results indicated that BbSwi6 and BbMbp1 localized in nuclei.

Yeast two hybrid (Y2H) assay was used to examine the interaction of BbSwi6 and BbMbp1. All constructs were valid as indicated by the yeast growth on the selective dropout (SD)-Leu-Trp medium. The mated transformants from the pair BbSwi6/BbMbp1 grew well on the SD-Leu-Trp-His-Ade medium, which indicating that BbSwi6 interacted with BbMbp1 directly (Figure 1B).

### 3.3. BbSwi6 Significantly Contributes to Vegetative Growth onNutrients

**Figure 2:**
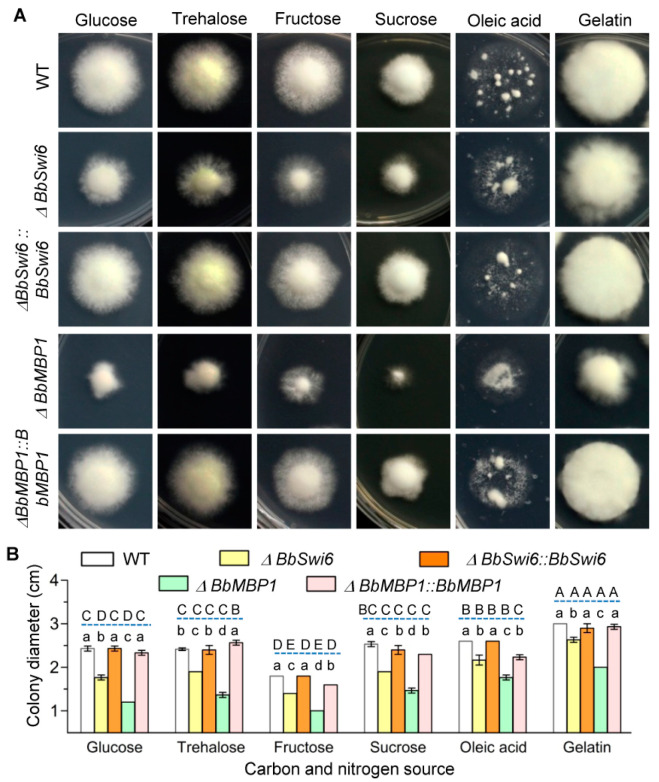
Effects of Swi6 loss on vegetative growth of *B. bassiana*. (**A**) On the basis of CzA medium, the carbon and nitrogen sources were replaced with the indicated nutrient. Blastopores of the indicated strain were prepared as the initial inocula and inoculated on plates. (**B**) After 7 d of incubation at 25 °C, the colony diameter was examined. The wild-type and Δ*BbMbp1* strains were used as the parallel controls. The different lowercaseletters show the significant difference among five strains at the indicated nutrient, and the capital letters indicate the significance among different nutrients for the indicated strain (Tukey’s HSD: *p* < 0.05).

As illustrated in Figure 2A,B, Δ*BbSwi6* mutant strain showed different growth trends on the minimal medium included different carbon and nitrogen sources. Colony diameters differed significantly among five strains (*F*_4,60_ = 1130.0, *p* < 0.01) andsix nutrients (*F*_5,60_ = 769.3, *p* < 0.01).Compared with the WT strain, the severe growth defects of Δ*BbSwi6* were observed on four carbon sources (glucose, trehalose, fructose and sucrose), and slight reduction in colony size of Δ*BbSwi6* occurred on oleic acid and gelatin. In addition, the *BbMbp1* loss led to more significant impairment in colony size on all tested nutrients. For example, on the medium with glucose as carbon source, the colony diameter of the WT strain was 2.43 ± 0.05 cm, and that of Δ*BbMbp1* was 1.20 ± 0 cm, which decreased 50.68%. The colony diameter of Δ*BbSwi6* was 1.77 ± 0.05 cm, which only decreased by 27.40% when compared with the WT strain.

### 3.4. BbSwi6 Is Required for Asexual Sporulation

**Figure 3:**
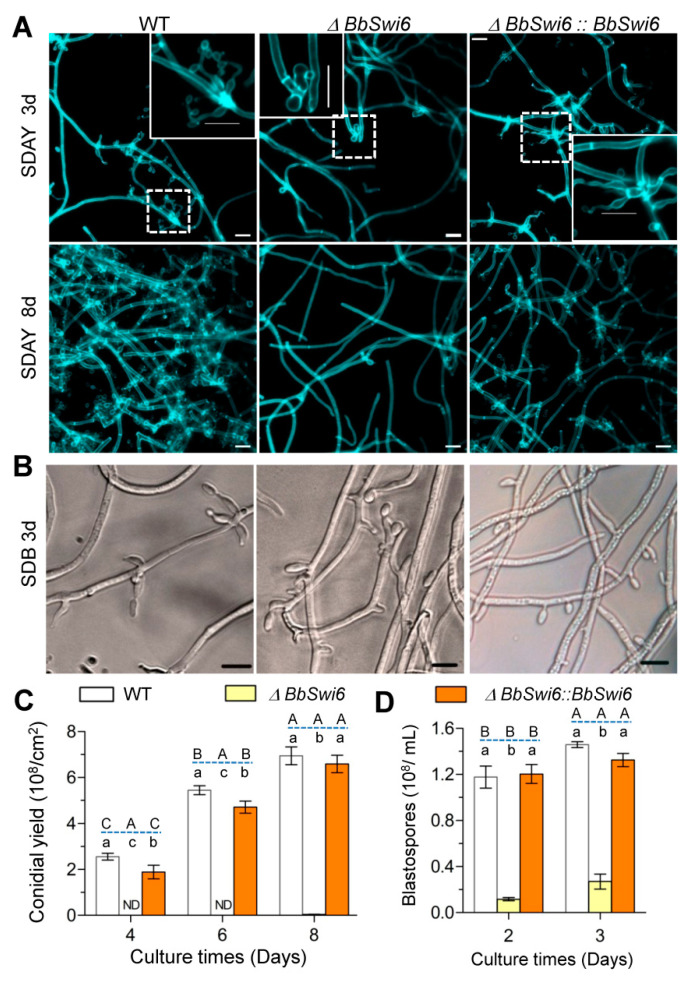
Phenotypic evaluation of asexual development. (**A**) The indicated strains were inoculated on the SDAY plates. Mycelia were sampled at 3 and 8 days post incubation (dpi). The wild-type (WT) and complementation (CP) strains generated conidiophores at 3 dpi and produced numerous conidia. However, Δ*BbSwi6* mutant did not form normal on conidiophores till 8 dpi. The enlarged conidiophores were shown. (**B**) In SDB medium, disruption of BbSwi6 caused a slight change in morphology of the blastospore-producing structures at 3 dpi. (**C**) Conidial production was determined at 8 dpi. “ND” means that no detectable conidia were observed. (**D**) Blastospore production was examined at 3 dpi. Gene loss significantly compromised the spore production on aerial surface and in submerged broth. The different lowercase letters show the significance among three strains at the indicated time point, and the capital letters indicate the significance among different time points for the indicated strain (Tukey’s HSD: *p* < 0.05). Scale bar: 10 µm.

On aerial surface (Figure 3A,C), the WT strains started conidiation at day 3 by formation of conidiophores and produced plenty of conidia at day 8 with a yield of 6.95 ± 0.32 × 10^8^ conidia/cm^2^. In contrast, Δ*BbSwi6* mutant strain, at 3 days post incubation, generated abnormal spore-producing structures. Even at day 8, only very few conidia were detected in Δ*BbSwi6* mutant strain. Its conidial yield was 0.03 ± 0.01 × 10^8^ conidia/cm^2^ and displayed a reduction of 99.51% when compared with the WT strain. There was significance in conidial yield among three strains (*F*_2,18_ = 1160.8, *p* < 0.01) andat three time points(*F*_2,18_ = 371.9, *p* < 0.01).

As shown in Figure 3B,D, in SDB medium, the WT and complemented strains generated numerous branch-like spore-formation structures on the mycelia. However, the blastospore-forming structures (morphology and number) were significantly changed in Δ*BbSwi6* mutant strain when compared with that of the WT strain. After 2 days of culture in SDB, Δ*BbSwi6* displayed a significantly reduced blastospore yield (0.116 ± 0.012 × 10^8^ spores/mL), whereas the WT and complemented strains generated 1.18 ± 0.08 and 1.20 ± 0.07 × 10^8^ spores/mL, respectively. Moreover, with the increase in culturing time, the yield gap between the WT and Δ*BbSwi6* strain decreased. On the second day, the yield of Δ*BbSwi6* decreased by 90.1% and on the third day, the decrease in yield was only 81.5%.

### 3.5. Disruption of BbSwi6Significantly Affected Fungal Pathogenicity

**Figure 4:**
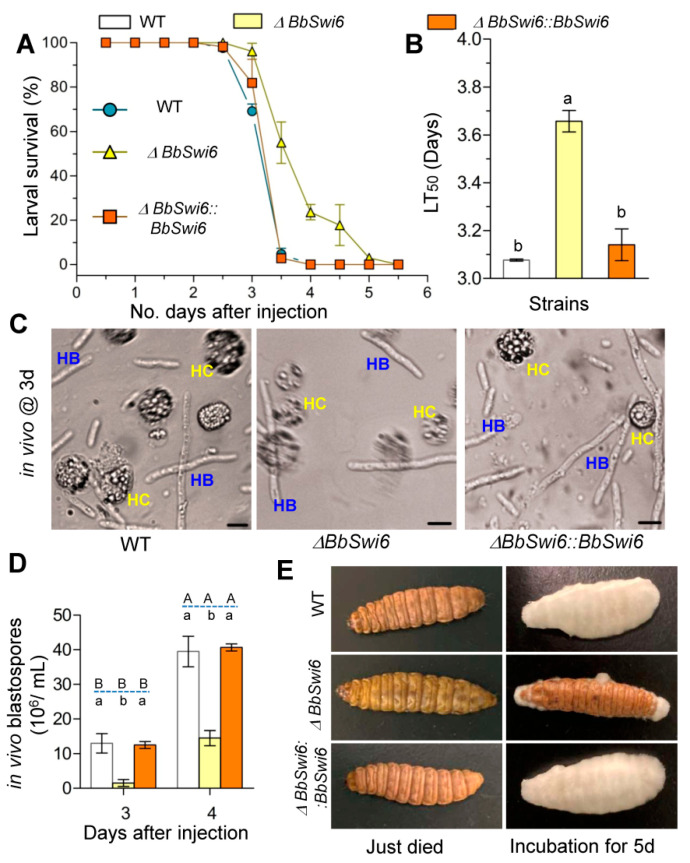
Fungal virulence. Blastopores of the indicated strain were prepared as the initial inocula. Spore suspension was injected into the hemoceol of *Galleria mellonella* larvae, and survival percentage was recorded daily. Compared with the wild-type strain (WT), Δ*BbSwi6* strain displayed a delayed survival curve (**A**) and an increased median lethal time (LT_50_) (**B**). Significant differences are designated the lowercase letters (Tukey’s HSD: *p* < 0.05). Gene loss impaired fungal dimorphism in the host hemoceol (**C**), and the production ofin vivoblastospores was significantly reduced (**D**). HB:in vivohyphal body. HC: insect hemocytes. (**E**) Δ*BbSwi6* strain displayed a significantly compromised ability to form mycoses on cadaver under moisture condition. The different lowercaseletters show the significant difference among five strains at the same time point, and the capital letters indicate the significance between two time points for the indicated strain (Tukey’s HSD: *p* < 0.05). Scale bar: 10 µm.

Injection bioassay was performed to examine the virulence of the Δ*BbSwi6* mutant against insect hosts. The accumulative mortality for Δ*BbSwi6* mutant did not significantly differ with that of the WT strain, but the mortality trend has been significantly delayed (Figure 4A). Cumulative mortalities for three strains were 100%. Δ*BbSwi6* mutant strain displayed an LT_50_ of 3.65 ± 0.036 d, with a delay of approximate 20%, when compared to those for the WT (3.07 ± 0.01 d) and complemented strain (3.14 ± 0.05 d) (Figure 4B) (*F*_2,6_ = 143.2, *p* < 0.01).In the host hemolymph, gene loss resulted in a significant reduction in vivo blastospores, although no morphological difference was found between the Δ*BbSwi6* mutant and the WT or complemented strain (Figure 4C). At 3d post infection, Δ*BbSwi6* mutant strain only produced 1.50 ± 1.00 × 10^6^ spores/mL, with an approximate 88% reduction, when compared with that of the WT strain (1.30 ± 0.27 × 10^7^ spores/mL). The spore yield of the Δ*BbSwi6* strain increased at 3 days post infection but still displayed an approximate 63.3% of WT (Figure 4D). After developing in moist boxes for 5 days, Δ*BbSwi6* mutant strain generated few mycelia on cadavers compared with the dense mycelia of the WT and complemented strains (Figure 4E). This result indicated that fungal virulence and growth on cadaver had been significantly impaired in Δ*BbSwi6* mutant.

### 3.6. BbSwi6 Mediates the Genome-Wide Expression during Fungal Differentiation

Disruption of *BbSwi6* significantly changed *B. bassiana* transcriptomes during conidial and blastospore formation. On aerial SDAY plates, the *BbSwi6* loss led to the altered expression of 1770 genes in which 783 genes were up-regulated (~7.6% of the genome) and 987 gene were down-regulated (~9.5% of the genome) (Table S2). In SDB medium (submerged condition), the *BbSwi6* transcriptome significantly differed with that of WT strain, which including 398 up-regulated genes (~3.8% of the genome) and 666 down-regulated genes (~6.4% of the genome) (Table S3).

**Figure 5:**
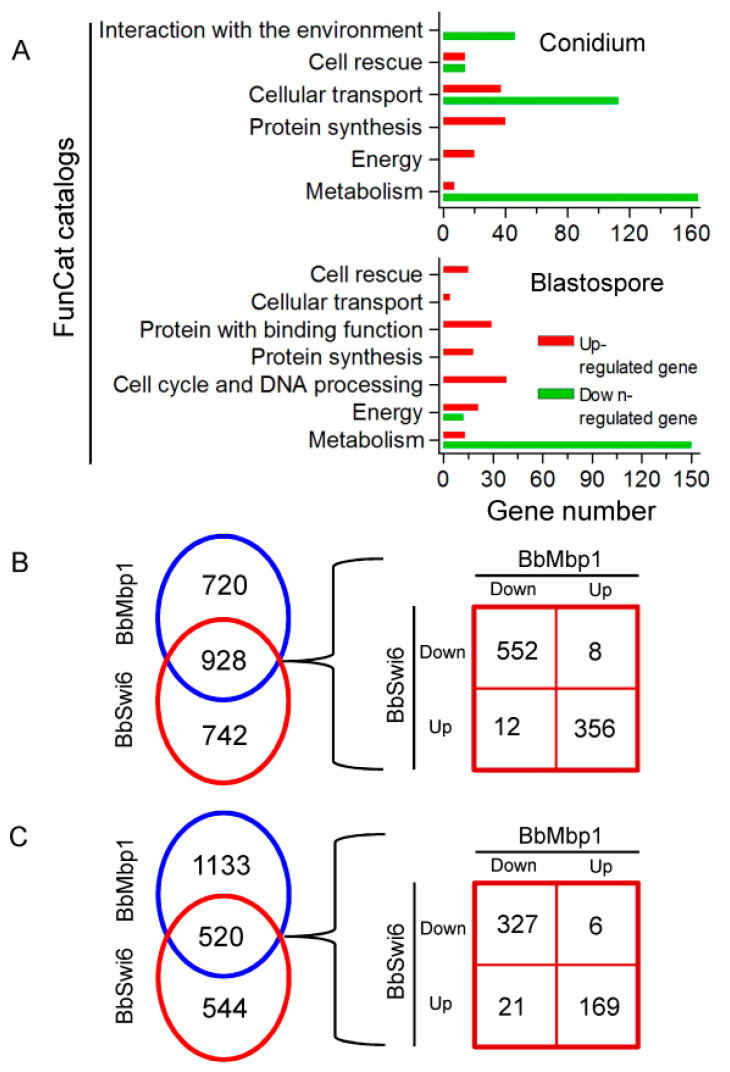
Functional distribution analyses of transcriptomic data. (**A**) Differentially expressed genes (DEGs) were individually determined in the Δ*BbSwi6* mutant via comparison with the wild-type strain during conidiation and blastospore formation. The up- and down-regulated DEGs were subjected to functional distribution analyses. BbSwi6 mediates different physiological terms in two types of asexual development. Red and green indicate the up- and down-regulated DEGs, respectively. Further, BbSwi6 and BbMbp1 differentially mediate the transcriptome during conidiation on aerial surface (**B**) or blastospore formation in broth (**C**).

Transcriptomic analyses indicated that *BbSwi6* losshad different effects during fungal differentiation on aerial surface and under submerged conditions (Figure 5A). Under aerial condition, the down-regulated genes (DRG) were enriched in four functional categories (i.e., metabolism, cell transport, cellular rescue and interaction with environment) (Figure 5A top panel and Table S4). The set of DRG represented in metabolism included a number of genes involved in nitrogen, aromate, vitamins and secondary metabolism. Enriched DRGs associated with cellular transport included a set of genes involved in transportation of heavy metal ion, amino acid, vitamins and so on. Repressed genes involved in cell rescue included many cytochrome P450 genes (detoxification pathway). Enriched DRGs involved in interaction with environment included a number of genes critical for homeostasis of metal ions and cellular sensing to external stimulus. The up-regulated genes (URG) in the mutant were largely enriched in metabolism, energy, protein synthesis, cell transport and cellular rescue (Figure 5A top panel and Table S5). For instance, the metabolism-associated URGs included a set of genes involved in deoxyribonucleotide metabolism. The energy-related URGs were involved in electron transport and aerobic respiration.

Under submerged condition, the DRGs were largely associated with metabolism and energy (Figure 5A bottom panel and Table S6). The metabolism-associated DEGs included genes involved in secondary metabolism (e.g., fructosyl amino acid oxidase, Cytochrome P450 and ubiquinone biosynthesis monooxygenase), biosynthesis of vitamin (e.g., torulene oxygenase and folylpolyglutamate synthase) and so on. The enriched categories for URGs involved metabolism, energy, cell cycle and so on, of which five categories (e.g., cell cycle and cellular rescue) were not enriched in the DRGs. The metabolism-associated URGs included the genes critical for the metabolism of amino acids (e.g., isoleucine, valine and leucine) (Figure 5A bottom panel and Table S7).

Compared with the *BbMbp1*-mediated transcriptome, the *BbSwi6*-mediated transcriptome had 928 and 520 differentially expressed genes under aerial (Figure 5B and Table S8) and submerged conditions (Figure 5C and Table S9), respectively. However, these genes showed significantly different distributions and expression patterns, and most overlapped genes were repressed in both gene disruption mutants.

## 4. Discussion

In yeast, transcriptional activator Swi6 plays a critical role in cell cycle and differentiation via recruiting Mbp1 and Swi4 to form the MBF and SBF complexes [30,31]. In filamentous fungus *B. bassiana*, a Swi6 ortholog was characterized. BbSwi6 contains three ANK domains. Our results demonstrate that BbSwi6 directly interacts with BbMbp1 in the nucleus, and plays important roles in growth, development and virulence in *B. bassiana*.

*BbSwi6* is significantly involved in fungal growth, development and virulence of *B. bassiana*. Compared with the phenotypes in Δ*BbMbp1* mutant strain [10], the *BbSwi6* loss has fewer influences. Filamentous fungi develop robust mycelia and conidiation process, which is essential for fungal survival and dispersal in ecosystem [32]. After killing the hosts, *B. bassiana* first grows intointense mycelia on cadavers and then produces numerous conidia which initiate the following infection cycle [33]. *BbSwi6* contributes to mycoses and conidiation on the dead hosts. *B. bassiana* conidia form and maturate on the ‘zig-zag’ conidiogenous cells [34]. *BbSwi6* is significantly required for formation of conidiogenous cells (Figure 3A). This result indicated that the loss of conidiation in Δ*BbSwi6* mutant strain might be due to the impaired conidiophore. *BbSwi6* mutation results in an approximate 99% reduction in conidial production (Figure 3C), and *BbMbp1* mutant lacks conidiation. As for blastospore formation under submerged condition, *BbSwi6* loss leads to 90% reduction in yield (Figure 3D), and disruption of *BbMbp1* causes 95% reduction [10]. As for fungal virulence, LT_50_ value for *BbSwi6* strain is delayed approximately 20%, and the *BbMbp1* loss results in a delay of 50%. Like most host-invasion myco-pathogens [35], *B. bassiana* undergoes dimorphic transition to generate single-cellular hyphal bodies, which is critical for fungal virulence [36]. Therefore, significantly weakened virulence might be caused by the dramatically compromised dimorphism in the host hemolymph. However, Δ*BbMbp1* mutant loses the dimorphism potential of 95% [10], and Δ*BbSwi6* mutant loses 88% (Figure 4D). These findings suggest that *BbMbp1* and *BbSwi6* might play different roles during fungal differentiation under aerobic and submerged conditions.

To verify this hypothesis, transcriptomic analyses were used to explore their impacts on global gene expression. Similar to *BbMbp1*, *BbSwi6* has broad influences on transcriptome during fungal development under aerial and submerged conditions; however, these two genes have different regulatory effects. During conidiation under aerobic condition, in terms of functional catalogs, *BbSwi6* performed transcriptional control of metabolism, cell transport, cellular rescue and interaction with the environment, whereas *BbMbp1* regulates conidial formation via transcriptional control of the cell cycle, metabolism and cell differentiation [10]. The BbSwi6- and BbMbp1-mediated transcriptomes have 39% DRGs overlapped. During conidiation, cell cycle is required for cell division. During fungal growth, cell cycle is required for septum formation and hyphal elongation [37]. *BbSwi6* and *BbMbp1* regulate the expression of Cdc14 which is a regulator of cyclin dependent kinase and contributes to conidiation [9]. Therefore, these two genes have positive roles in vegetative growth. In addition, *BbMbp1* contributes to the expression of a hydrophobin which localizes on the conidial surface of *B. bassiana* [38] and significantly contributes to conidiation process [39]. BbSwi6 and BbMbp1 arerequired for transcription of Ca^2+^/calmodulin-dependent protein kinase (CaMK) which mediates signaling transduction in a plenty of cellular processes, and plays an important role in conidiation in filamentous fungus (e.g., *Magnaporthe oryzae* and *Arthrobotrys oligospora*) [40,41]. These findings suggest that *BbSwi6* and *BbMbp1* still have common effects on the conidiation-related genes, though they execute divergent transcriptional regulation on different functional categories.

During dimorphism under submerged condition, *BbSwi6* also performs comprehensive transcriptional regulation. On the basis of functional categories, *BbSwi6* contributes to blastospore generation by regulating the genes involved in metabolism and energy (Figure 5A). The energy metabolism has been linked with dimorphism in filamentous fungi. In *Talaromyces* *marneffei*, bioinformatic evidence indicates that energy metabolism is urgent during switching from mycelia form to yeast form [42]. In *Mucor circinelloides*, Arf-like proteins are involved in maintaining mitochondrial number homeostasis which is required for energy supply [43]. These results suggest that energy metabolism is the evolutionally conserved requirement for dimorphic transition in filamentous fungi. Nevertheless, to explore the mechanisms involved in transcriptional regulation, more experiments are needed. During blastospore formation, *BbMbp1* mediates transcriptional control of the genes related to metabolism, cell transport and development [10]. The functional catalog of metabolism is shared by the BbSwi6- and BbMbp1-mediated transcriptomes (Figure 5B). The linkage of metabolism with dimorphism has been revealed in filamentous fungi (e.g., *Penicillium marneffei* and *Ustilago maydis*) [44,45]. In addition, these two genes are required for expression of the member of heat shock protein (Hsp) family (e.g., DnaJ protein). DnaJ protein is also known as Hsp40. *B. bassiana* Mas5 is homologous to DnaJ and critical for fungal blastospore production [46]. *BbSwi6* and *BbMbp1* contribute to the transcriptional activation of mitogen-activated protein kinase (MAPK) gene Sty1 (Table S9). MAPK cascade plays an essential role in *B. bassiana* development, including fungal dimorphic transition into blastospores [47]. This implies that *BbSwi6* and *BbMbp1* have a common regulation on the critical genes and pathways during blastospore formation, although they perform divergent roles on different functional groups of genes.

## 5. Conclusions

All in all, BbSwi6 interacts with BbMbp1 in nuclei and forms the transcription complex MBF. BbSwi6 plays a vital role in fungal growth, differentiation and virulence in *B. bassiana*. Although they work as components of MBF, BbSwi6 and BbMbp1 mediate significantly different global expression patterns during fungal differentiation into conidia and blastospores in *B. bassiana*.

## Data Availability

The data presented in this study are openly available in GEO database. GEO reviewer link: Go to https://www.ncbi.nlm.nih.gov/geo/query/acc.cgi?acc=GSE171375 accessed on 6 September 2020. Enter token ‘kpgxsaokdxodfcf’ into the box.

## Supplementary Materials

The following are available online at https://www.mdpi.com/article/10.3390/jof7060411/s1, Figure S1: Construction of gene disruption and complementation mutant strains; Table S1: Primers used in this study; Table S2: Differentially expressed genes (DEGs) in comparison of wild type/Δ*BbSwi6* during conidiation process; Table S3: Differentially expressed genes (DEGs) in comparison of wild type/Δ*BbSwi6* during blastospore formation; Table S4: FunCat analysis of the down-regulated DEGs during conidiation; Table S5: FunCat analysis of the up-regulated DEGs during conidiation; Table S6: FunCat analysis of the down-regulated DEGs during blastospore formation; Table S7: FunCat analysis of the up-regulated DEGs during blastospore formation; Table S8: DEGs in the paired comparison between the BbSwi6- and BbMbp1-mediated transcriptomes during conidiation; Table S9 DEGs in the paired comparison between the BbSwi6- and BbMbp1-mediated transcriptomes during blastospore formation.
